# Impact of prenatal LPS on sepsis-related neurobiological outcomes

**DOI:** 10.1007/s11011-026-01852-6

**Published:** 2026-05-19

**Authors:** Fernanda Frederico Gava, Larissa Joaquim, Naila Maciel, Khiany Mathias, Richard Simon Machado, Brenno Farias, Thainá Cidreira, Sabini Abrahão, Beatriz Steiner Cardoso, Marina Goulart, Carolina Giassi Alano, Rafaela Tezza Matiola, Isabela da Silva Lemos, Rafael Mariano de Bitencourt, Jaqueline da Silva Generoso, Emilio Luiz Streck, Fabricia Petronilho

**Affiliations:** 1 Laboratory of Experimental Neurology, Graduate Program in Health Sciences, University of Southern Santa Catarina (UNESC), Criciúma, SC Brazil; 2 Laboratory of Neurobiology of Inflammatory and Metabolic Processes, Graduate Program in Health Sciences, Health Sciences Unit, University of South Santa Catarina, Tubarão, SC Brazil; 3 Behavioral Neuroscience Laboratory, Postgraduate Program in Health Sciences, University of Southern Santa Catarina, Tubarão, Santa Catarina Brazil; 4 Laboratory of Experimental Biomedicine, Graduate Program in Health Sciences, University of Southern Santa Catarina, Criciúma, SC Brazil

**Keywords:** Sepsis, Sex differences, Prenatal immune challenge, Lipopolysaccharide, Neuroinflammation, Oxidative stress, Mitochondrial dysfunction

## Abstract

Background early-life challenges during the prenatal period can induce persistent neurological alterations. The immature innate immune system exhibits plasticity, and appropriately timed stimuli may shape immune responses into adulthood. Objective to investigate whether prenatal exposure to lipopolysaccharide (LPS) modulates brain alterations after sepsis in adult offspring of both sexes. Methods pregnant Wistar rats received an intraperitoneal injection of LPS (100 µg/kg) on gestational day 9.5. In adulthood, offspring underwent cecal ligation and perforation (CLP) or sham surgery, generating four groups: sal+sham, LPS+sham, sal + CLP, and LPS + CLP. Survival was monitored, and behavioral assessments (open field habituation and forced swim test) were performed 10 days post-surgery. Brain regions (cortex, prefrontal cortex, hippocampus) were analyzed for cytokine levels, myeloperoxidase (MPO) activity, nitrite/nitrate (N/N) concentrations, oxidative damage, catalase (CAT) activity, and mitochondrial respiratory chain complex activities (I, II, IV). Results prenatal LPS improved survival, enhanced habituation memory (both sexes), and reduced depressive-like behavior (females). It increased IL-10 across brain regions and IL-6 in the male hippocampus, while reducing N/N in the female prefrontal cortex and hippocampus. Oxidative damage was attenuated, with reduced lipid peroxidation in the male prefrontal cortex and decreased protein carbonylation in both sexes. CAT activity increased in the male cortex and hippocampus. Prenatal LPS preserved mitochondrial complex I and IV activities in the male hippocampus, complex I in cortex and prefrontal cortex, and complex II in the female prefrontal cortex. Conclusion prenatal immune challenge with LPS confers sex-dependent neuroprotective effects, reducing inflammation, oxidative damage, and mitochondrial dysfunction induced by severe sepsis in adult offspring.

**Graphical Abstract:**

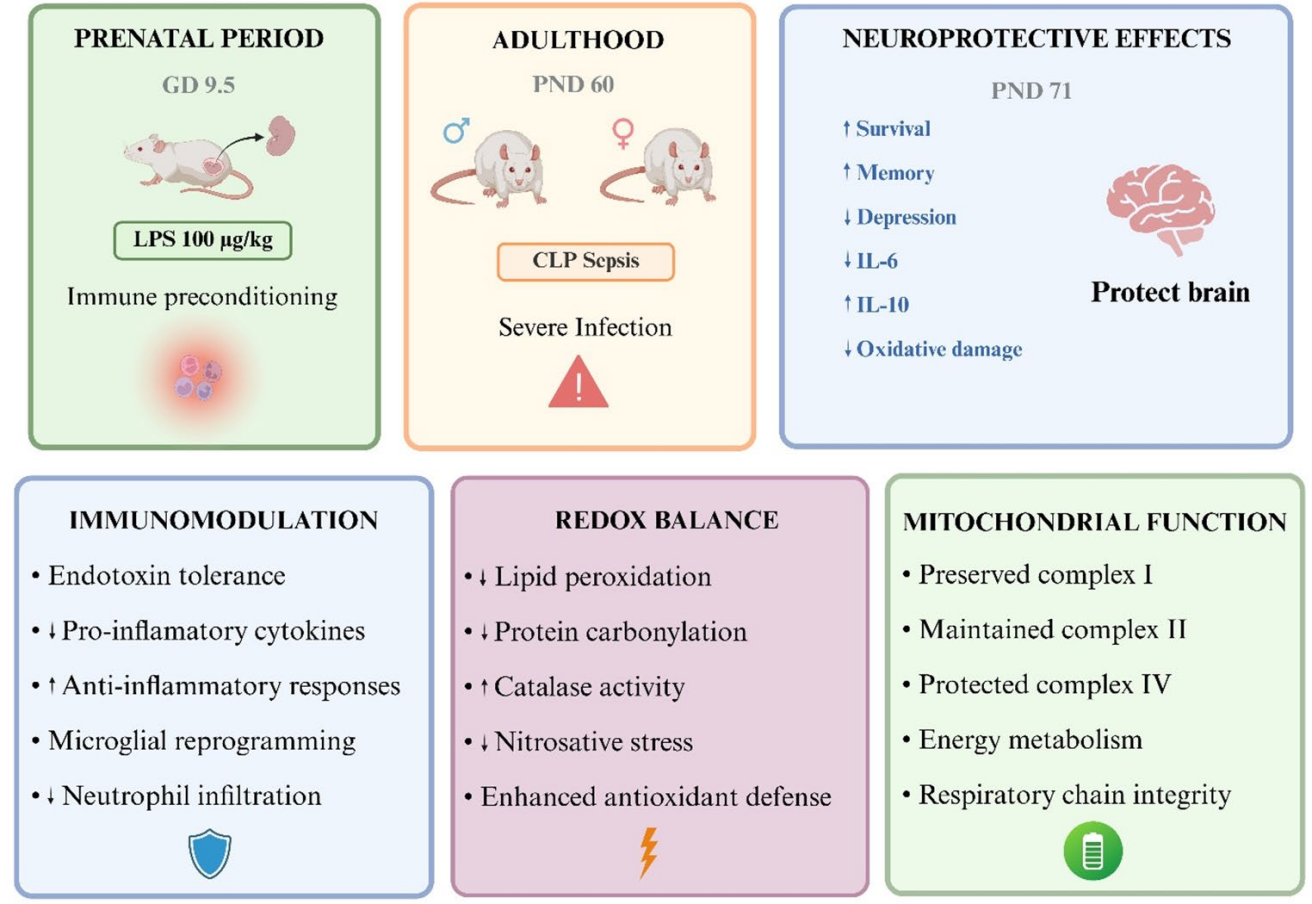

## Introduction

Sepsis is defined by the International Consensus (Sepsis-3) as “life-threatening organ dysfunction caused by a dysregulated host response to infection,” highlighting the central role of inflammatory and immune imbalance in its pathogenesis (Singer et al. 2016). Globally, sepsis affects approximately 49 million people each year, with mortality reaching up to 60% in cases of septic shock (Vincent et al. 2019; Rudd et al. 2020) At the cellular and molecular levels, sepsis involves a complex cascade including excessive cytokine release, immune suppression, mitochondrial dysfunction, coagulopathy, endocrine dysregulation, and oxidative damage, ultimately contributing to multiorgan failure (Petronilho et al. 2016; Della Giustina et al. 2017a; Danielski et al. 2018a; Huang et al. 2019). 

Innate immune activation begins with macrophage recognition of pathogen-associated molecular patterns (PAMPs) and damage-associated molecular patterns (DAMPs), triggering transcriptional programs driven by pattern recognition receptors (PRRs) (Mehta et al. 2020; Coperchini et al. 2021; Wang et al. 2025). This overwhelming systemic inflammation can rapidly affect the central nervous system (CNS), resulting in sepsis-associated encephalopathy (SAE), a complication observed in up to 70% of patients (Fu et al. 2014; Andonegui et al. 2018; Michelon et al. 2020). SAE is characterized by endothelial injury, blood–brain barrier (BBB) dysfunction, glial activation, oxidative stress, and neuronal apoptosis, particularly in vulnerable brain regions such as the hippocampus and prefrontal cortex (Iwashyna et al. 2012; Biff et al. 2013; Sonneville et al. 2013, 2017; Catalão et al. 2020a; Moraes et al. 2021; Simon Machado et al. 2023; Mathias et al. 2024; Li et al. 2025). Persistent neuroinflammation alters neurotransmission, neuroendocrine circuits, and cognitive function, contributing to long-term neurological deficits in survivors (Pan et al. 2018; Peeters et al. 2018; Alam et al. 2020; Gu et al. 2021; Badawy et al. 2021). Because of the brain’s high metabolic activity and relatively low antioxidant defenses, mitochondrial dysfunction and oxidative damage play critical roles in SAE pathogenesis (Danielski et al. 2018c; de Mello et al. 2019; Nguyen et al. 2023; Zong et al. 2024). 

Although most studies focus on how sepsis induces brain injury, an emerging body of evidence suggests that immune responses to infection are strongly shaped by developmental history. Early-life periods—including the intrauterine environment—represent windows of heightened plasticity in the innate immune system (Danese and Lewis 2017; Hong and Medzhitov 2023). Stimuli occurring during gestation can induce long-lasting modifications in immune maturation, neurodevelopment, and stress-responsiveness that persist into adulthood. In contrast, aging is marked by progressive immune decline and increased susceptibility to infections (Cisneros et al. 2022). 

Prenatal immune activation (PIA), frequently modeled by exposure to lipopolysaccharide (LPS), is traditionally associated with detrimental outcomes, including altered neurodevelopment, increased vulnerability to neuropsychiatric disorders, and dysregulated cytokine responses later in life. However, more recent studies demonstrate that the effects of PIA are context-dependent and influenced by factors such as timing, dose, maternal environment, and fetal sex. Several reports show that prenatal exposure to microbial mimetics can induce immune training or tolerance, enhancing the capacity to mount balanced inflammatory responses upon later challenges. For example, offspring of LPS-exposed dams exhibit enhanced Th1 cytokine responses, altered regulatory pathways, and persistent changes in microglial and mitochondrial programming into adulthood (Kwon et al. 2022; Hofsink et al. 2024; Euclydes et al. 2024).

These observations introduce a critical conceptual shift: prenatal immune activation can act not only as a risk factor but also as a developmental immune modulator, potentially increasing resilience to systemic inflammatory insults in later life. The possibility that PIA establishes a tolerogenic or primed state capable of modulating adult responses to severe infections such as sepsis remains largely unexplored, particularly regarding CNS vulnerability.

Despite advances in understanding sepsis pathophysiology, the long-term consequences of maternal immune activation on offspring susceptibility or resilience to sepsis-induced brain dysfunction are poorly understood. This gap is especially relevant given the recognized sex differences in immune, inflammatory, and mitochondrial responses across the lifespan, which may influence sepsis outcomes and neuroinflammatory trajectories.

Therefore, this study aimed to investigate whether prenatal exposure to LPS modulates neuroinflammatory, oxidative, behavioral, and mitochondrial alterations induced by sepsis in adult offspring of both sexes.

We hypothesized that prenatal LPS would induce a long-lasting immunomodulatory state capable of attenuating sepsis-induced brain dysfunction in adulthood, in a sex-dependent manner.

## Materials and methods

### Animals

Adult male and female Wistar rats were obtained at 30 days of age from the Bioterium of the Federal University of Santa Catarina (UFSC), housed and acclimatized for 30 days in the Bioterium of the University of Southern Santa Catarina (UNISUL), and were therefore 60 days old (weighing 250–350 g) at the beginning of the experimental procedures. 

**Fig. 1:**
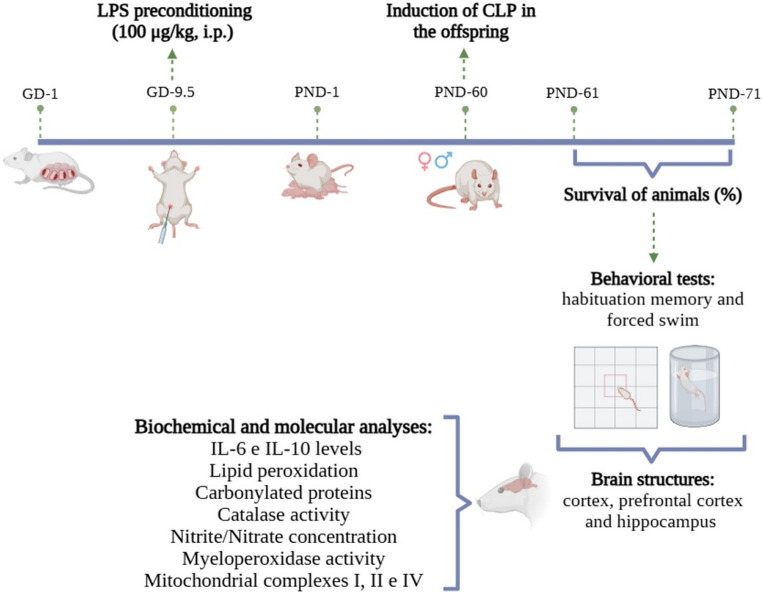
Experimental design. Schematic representation of the experimental protocol. Pregnant dams received lipopolysaccharide (LPS, 100 μg/kg, i.p.) or saline on gestational day (GD) 9.5. Offspring were subjected to cecal ligation and puncture (CLP) or sham surgery on postnatal day (PND) 60. Survival was monitored from PND 61-71. On PND 71, behavioral tests (habituation memory and forced swim test) were performed, followed by biochemical and molecular analyses (IL-6 and IL-10, lipid peroxidation, protein carbonylation, catalase, nitrite/nitrate, myeloperoxidase, and mitochondrial complexes (I, II, and IV) in the cortex, prefrontal cortex, and hippocampus

The experiment was divided into two stages (Fig. 1). In stage 1, ten adult male rats and twenty adult female rats, aged 60 days and weighing between 250 and 300 g, were used exclusively for mating to obtain the litters. After confirmation of pregnancy, the dams were randomly assigned to receive either saline (Sal) (control) or LPS, forming the two maternal groups for the maternal immune activation protocol. In stage 2, the offspring generated in stage 1 were used, consisting of 32 males and 32 females aged 60 days and weighing between 250 and 350 g. These animals were allocated into four experimental groups according to maternal treatment and adult surgical condition: (1) Sal+Sham, (2) LPS+Sham, (3) Sal + CLP, and (4) LPS + CLP. Each combination was evaluated separately for males and females, consisting of 8 animals per sex and group. Specifically, no more than one male and one female from each litter were allocated to the same experimental group, ensuring that the litter rather than the individual offspring was treated as the true experimental unit, as recommended by Lazic et al. (2018). Offspring were randomly assigned across the four experimental conditions (Sal+Sham, Sal + CLP, LPS+Sham, and LPS + CLP), thereby guaranteeing independent sampling and minimizing bias associated with shared genetic and early environmental factors. Furthermore, each experimental group included offspring derived from at least six distinct litters, and no group contained more than one same-sex pup from the same litter. This approach ensures that the variability observed reflects true between-litter differences, preventing pseudoreplication arising from treating littermates as independent samples. Due to the intrinsic mortality associated with the CLP model, the final number of animals varied across experimental endpoints. Behavioral analyses were performed using all surviving animals at day 10 post-CLP. For biochemical, immunological, and mitochondrial analyses, a predefined subset of animals (approximately *n* = 6–8 per group) was used, based on tissue availability and technical requirements. The exact sample size for each analysis is specified in the corresponding figure legends. The animals from both stages were placed in polypropylene boxes (41 × 34 × 16 cm), with wood shavings, with 5 rats per box, under a 12-hour light/dark cycle (06:00 to 18:00), controlled by timer, receiving food and water ad libitum in an air-conditioned environment. The ambient temperature was maintained at 24 °C ± 1 °C. All experimental procedures were carried out in accordance with international recommendations for the care and use of laboratory animals. The protocol was approved by the Ethics Committee for the Use of Animals of the University of Southern Santa Catarina, under protocol number 20.021.4.01.IV. 

## Experimental design

### LPS administration

The matrices were randomly divided into two groups according to the experimental protocol. One group received a single intraperitoneal (IP) injection of lipopolysaccharide (LPS) extracted by phenolic method from Escherichia coli serotype O127:B8 (Sigma-Aldrich® LPS: Cat. No. L3129), at a dose of 100 µg/kg on gestational day (GD) 9.5 (9 days and 12 h), diluted in sterile 0.9% NaCl (50 µg/mL). This dose is widely used to induce mild maternal immune activation without causing fetal resorption or maternal toxicity, while being sufficient to alter fetal immune programming (Carlezon et al., 2019; Potter et al., 2023), characterizing the LPS-exposed experimental group.The other group received only sterile 0.9% NaCl IP solution in the volume equivalent to the experimental group, characterizing the control group (sal-exposed). All injections were performed in the afternoon (3:00–5:00 pm), during the period corresponding to gestational day (GD) 9.5. Based on mating confirmation at 00:00 (GD 0), GD 9.5 corresponded to the interval ending at 12:00 pm on the ninth day (da Rosa et al. 2022). The births occurred naturally, and no handling of the litters was performed on postnatal days (PND) 1 and 2 to avoid disrupting maternal care or triggering maternal rejection. Initial sex identification was conducted on PND 3 by visual inspection of the anogenital distance, which is markedly greater in males. Between PND 8 and 15, sex differentiation was confirmed by the presence of descended testes in males and visible nipples in females. The study included male and female offspring from dams exposed to LPS or sal during the prenatal period. Offspring remained with their mothers until weaning on PND 21. 

### Sepsis induction

At 60 days of age, male and female offspring were subjected to intra-abdominal sepsis induction using the CLP technique, as described by Hubbard et al. (2005). Briefly, animals were anesthetized intraperitoneally (i.p.) with ketamine hydrochloride (80 mg/kg) (Syntec, Cat. No. 0407001) and xylazine hydrochloride (10 mg/kg) (Syntec, Cat. No. 0406001), a 3-cm midline laparotomy was performed to expose the cecum and adjoining intestine. The cecum was tightly ligated with a 3.0 silk suture at 50% below the ileocecal valve and perforated once with a 14-gauge needle to induce a mid-grade sepsis. The cecum was squeezed gently to extrude a small amount of feces through the perforation site. The cecum was then returned to the peritoneal cavity, and the laparotomy was closed with 3.0 silk sutures. All animals received basic support (NaCl 0.9%; 50 mL/kg) and antibiotic (ceftriaxone; 30 mg/kg) (Cristália, Cat. No. 010208) subcutaneously (s.c.) immediately after and 12 h after CLP. In the sham operated group, the rats were submitted to all surgical procedures but the ligation and puncture of the cecum. To minimize variability between different experiments, the CLP procedure was always performed by the same investigator.

### Survival

For 10 days after induction of sepsis by CLP, the survival of the animals was evaluated. Immediately after the survival assessment, the animals were subjected to behavioral tests to assess memory and learning, as well as assessment of depressive-type behavior.

### Behavioral tests

Behavioral tests were carried out in 10 days after sepsis induction by CLP. The tests began at 10:00, which were monitored and recorded using a webcam attached to notebooks. Furthermore, the animals were previously acclimatized in a reserved room in the bioterium for a period of 60 min, representing the period of habituation to the environment. Behavioral testing was performed in two sessions. The open field test was conducted first, and the forced swim test was performed 24 h later to avoid carryover effects between procedures. 

The open field habituation test was carried out in an apparatus consisting of a box measuring 40 cm × 60 cm in an open field surrounded by a 50 cm wall made of wood with a glass front. The open field floor is divided into 9 rectangles by black lines. The test was divided into two stages, in the first stage called habituation (training), the animals were placed in the apparatus so that they could explore the environment. The exploration time was 5 min, in which the number of crossings (the moment the animal crosses the line) was counted. After 24 h, the test was carried out, where the same animal was placed again for 5 min in the apparatus and the same parameter was evaluated. Habituation memory is then assessed, since animals are expected to remember the environment, causing them to explore the apparatus environment less(Izquierdo and Medina 1997; Taubenfeld et al. 1999; Cammarota et al. 2000; Fu et al. 2017).

The forced swimming test was performed according to the description. This test involves two sessions called training and testing, carried out in a cylindrical tank filled with water at a temperature of ± 26 °C and deep enough for the animal not to be able to touch the bottom of the tank or escape. In the training session, the animal is placed in the water and allowed to swim freely for 15 min. 24 h after training, the animal is returned to the tank for the test session. In this session, the time that the animal remains motionless in the water, without the intention of swimming (immobility) for 5 min, is counted. This behavior is a representation of demotivation (Porsolt et al. 1977).

### Biochemical, immunological and molecular analysis

After carrying out the last behavioral test, the animals were submitted to a euthanized, through an overdose of sodium thiopental (0.5 g/kg via IP). The brains were removed and dissected into the cortex, frontal cortex and hippocampus and, immediately after, they were stored in liquid nitrogen to preserve the samples. At the end of preparation, the samples were organized in boxes and stored at − 80 °C for biochemical, immunological and molecular evaluations.

### Quantification of total proteins

Biochemical analyzes were normalized for protein content, which was measured according to the method of Lowry and colleagues (Lowry et al. 1951). Solutions of 2% sodium carbonate in 0.1 mol L-1 sodium hydroxide were used; copper sulfate 1%; sodium and potassium tartrate 2%; solution containing 2% sodium carbonate, 0.01% copper sulfate and 0.02% potassium tartrate in 0.1 mol L-1 sodium hydroxide and the phenol reagent FolinCiocalteau diluted in a 1:1 ratio (v/v) in type 1 ultrapure water. To perform the standard curve, bovine serum albumin (Sigma-Aldrich^®^, Cat. No. A9647, RRID: AB_2890960) was used.

### Inflammatory parameters

The quantification of IL-6 and IL-10 cytokines was carried out using an enzyme-linked immunosorbent assay (ELISA), following the instructions in the leaflets of the commercial kits (Sigma-Aldrich^®^, Cat. No. RAB0311 and RAB0247). Brain samples were obtained from the cortex, frontal cortex, and hippocampus of the offspring. All samples and standards were analyzed in duplicate to ensure measurement reliability.

Initially, in a 96-well plate, 100 µl/well of the blank, standard, or samples were added, and the plate was incubated for 2 h at room temperature on a Kline-type shaker at low speed. After this period, 100 µl of detection reagent A was added and incubated for 1 h at 37 °C. After incubation, the plate was washed three times with the washing solution and, immediately after, 100 µl of reagent B was added to each well, followed by a 30-minute incubation. Afterwards, the washing procedure was carried out five times, 90 µl of 3,3′,5,5′-tetramethylbenzidine was added to each well and the plate was incubated for 20 min at 37 °C, generating a blue color. 

At the end of the incubation, a reaction stopping solution was added, and absorbance was measured using a microplate reader at 450 nm. Cytokine concentrations were calculated using a standard curve generated from serial dilutions of the provided standards on each plate. The unit of measurement used was pg/ml.

### Evaluation of myeloperoxidase (MPO) activity

The tissue was homogenized (50 mg/mL) in 0.5% of hexadecyltrimethylammonium bromide (Sigma-Aldrich^®^, Saint Louis, USA – Cat. No H5882) and centrifuged. The suspension was homogenized, and an aliquot of supernatant was mixed with a solution of 1.6 mM 3,3′,5,5′-Tetramethylbenzidine (TMB) Sigma-Aldrich^®^, Cat. No T0440 and 1 mM H2O2. The MPO activity was measured spectrophotometrically at 650 nm at 37 °C. The results were expressed as mU/mg of protein(Young et al. 1989).

### Oxidative stress parameters

As an index of oxidative damage to lipids, we verified the formation of thiobarbituric acid reactive species (TBARS) in an acid-heating reaction, as previously described (Draper and Hadley 1990). Briefly, the samples were mixed with 1 mL of 10% trichloroacetic acid and 1 mL of 0.67% thiobarbituric acid, and then heated in a boiling water bath for 15 min. TBARS was determined by the absorbance at 535 nm using 1,1,3,3-tetramethoxypropane as an external standard. The results were expressed as malondialdehyde equivalents nmol/mg of protein.

The protein oxidative was assessed by the determination of carbonyl groups based on the reaction with dinitrophenylhidrazone, as previously described (Levine et al. 1990). Briefly, proteins were precipitated by the addition of 20% trichloroacetic acid and dissolved in dinitrophenylhidrazone, and the absorbance was read at 370 nm. The results were expressed as protein carbonylation nmol/mg of protein.

Catalase (CAT; EC 1.11.1.6) activity was determined by the absorbance decrease at 240 nm in a reaction medium containing 20 mM H2O2, 0.1% Triton X-100, 10 mM potassium phosphate buffer, pH 7.0, and the supernatants containing 0.1–0.3 mg protein. mL-1(Aebi 1984). The specific activity was expressed as mU/mg of protein.

Nitrite/nitrate (N/N) concentration was measured by Griess reaction in the samples by adding 100 µl of Griess reagent 0.1% (w/v) naphthylethylendiamide dihydrochloride in H2O and 1% (w/v) sulphanilamide in 5% (v/v) concentrated H3PO4, vol. 1:1 to the 100 µl sample. After one hour of incubation at room temperature, absorbance was recorded in a spectrophotometer at 550 nm (Green et al. 1982). The results were expressed as nmol of N/N/mg of protein.

### Evaluation of mitochondrial complex activity

Cortical, frontal cortex, and hippocampal tissues were homogenized in SETH buffer (250 mM sucrose, 2 mM EDTA, 10 mM Tris base, and 50 IU/mL heparin; pH 7.4). The homogenate was centrifuged at 3,000 RPM for 10 min, and the resulting supernatant was stored at − 80 °C until analysis. Complex I activity was determined in a medium containing potassium phosphate buffer (100 mM, pH 7.4) and homogenate proteins, to which NADH (14 mM), rotenone (1.0 mM), and ferricyanide (10 mM) were added. Absorbance was recorded for 3 min at 420 nm at 25 °C, and enzyme activity was calculated based on the NADH-dependent reduction of ferricyanide, reflected by a decrease in absorbance (Cassina & Radi, 1996). Complex II activity was assessed in an incubation medium composed of potassium phosphate buffer (40 mM, pH 7.4), sodium succinate (16 mM), and DCIP (8 µM), together with homogenate proteins, incubated at 30 °C for 20 min. Sodium azide (4 mM) and rotenone (7 mM) were then added, and the reaction was initiated by adding DCIP (40 mM). Absorbance was measured for 5 min at 600 nm, and complex II activity was expressed as the decrease in absorbance resulting from DCIP reduction (Fischer et al.  1985). For complex IV activity, an incubation medium containing potassium phosphate buffer (10 mM, pH 7.0), dodecyl maltoside (0.6 mM), and homogenate proteins was used, and the reaction was initiated by adding reduced cytochrome c (0.7 µg). Cytochrome c oxidation was monitored for 10 min at 25 °C, with readings taken at 550 nm. Complex IV activity was determined based on the decrease in absorbance corresponding to the oxidation of reduced cytochrome c (Rustin et al. 1994).

### Statistical analysis

To evaluate data distribution, the Shapiro-Wilk normality test was applied. The variables were presented as mean ± standard deviation, when the distribution is symmetrical, or as median, when it is asymmetrical. One-way ANOVA or two-way ANOVA analysis of variance was performed, followed by Tukey post hoc for multiple comparison of values with symmetric or normal distribution. The Kruskal-wallis test was used followed by Dunn’s post hoc test when the variables had an asymmetric distribution. For behavioral data, analysis was performed using the Student’s t test, for symmetrical data, or the Mann-Whitney test, when the data were asymmetrical. For the survival test it was evaluated by Kaplan Meier. For all analyses, a 95% confidence interval (*p* ≤ 0.05) was adopted. All analyzes were performed using the Statistical Package for the Social Sciences (SPSS) version 20. The graphs were generated using the Prism 9 software (GraphPad©).

## Results

### CLP-induced mortality and the protective influence of prenatal LPS exposure

**Fig. 2:**
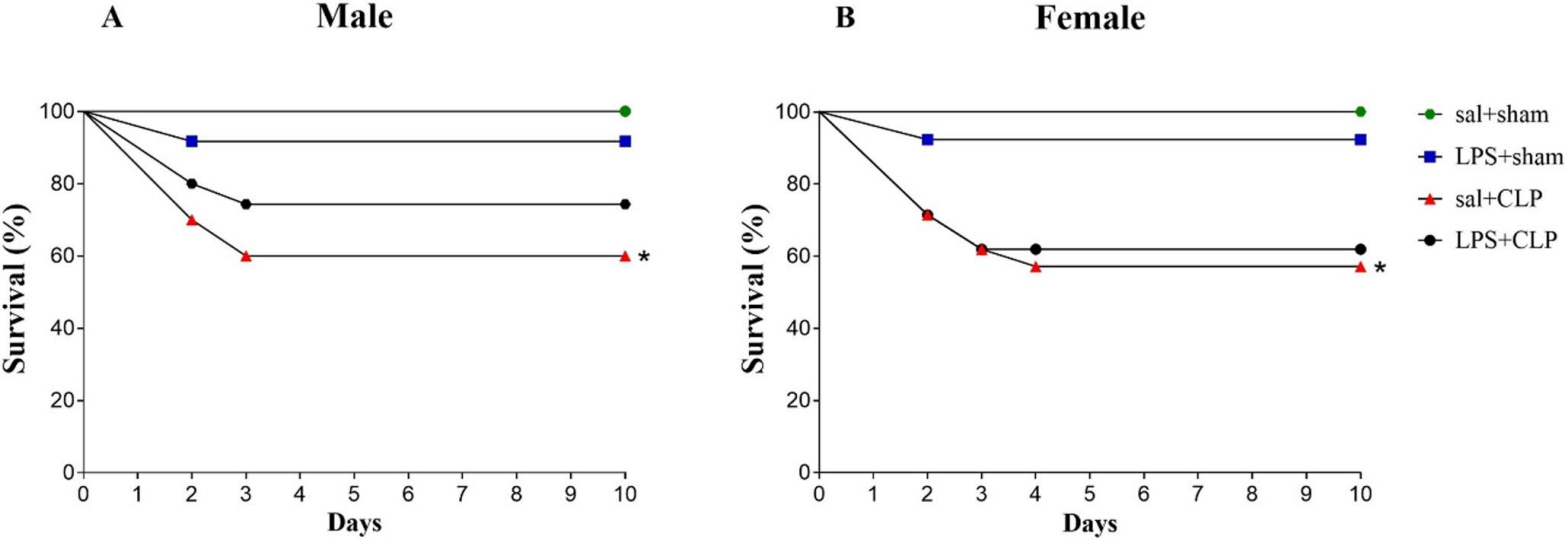
Survival curves. Effects of prenatal LPS preconditioning on survival of rats male (**A**) and female (**B**) subjected to CLP-induced sepsis in adulthood (*n* = 10-12/group). Experimental groups: sal+sham, sal+CLP, LPS+sham, and LPS+CLP. Survival was monitored daily for 10 days (PND 61-71). Data expressed as survival percentage. Statistical analysis: Kaplan-Meier survival curve with log-rank test. *p < 0.05 vs. sal+sham

Survival analysis indicated that CLP surgery substantially reduced survival in both sexes, with males exhibiting 60% survival (Fig. 2A) and females 57% (Fig. 2B), relative to the 100% survival observed in the sal+sham group. Notably, survival in the LPS+sham and LPS + CLP groups remained comparable to that of the sal+sham group across sexes, with no statistically significant differences. 

### Prenatal LPS enhances resilience to CLP-induced impairments in habituation memory

**Fig. 3:**
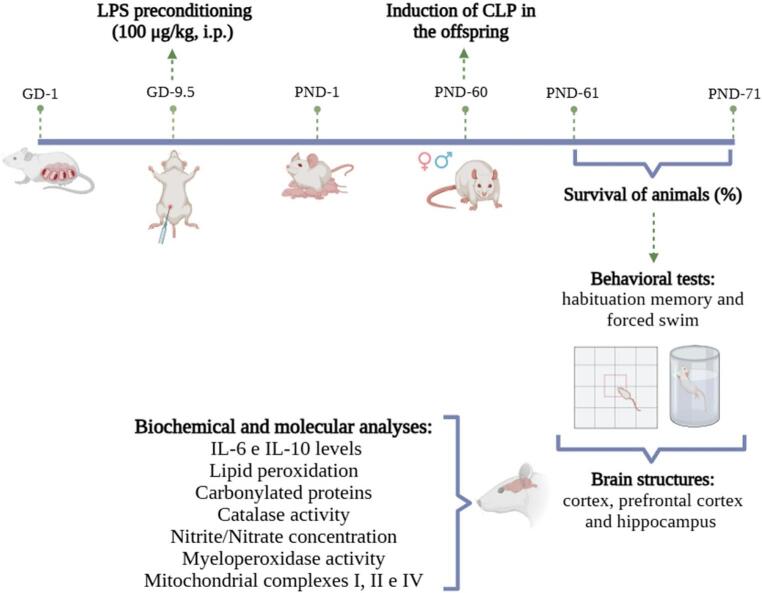
Behavioral assessments: habituation memory and forced swim test. Effects of prenatal LPS preconditioning on habituation memory (**A**-**B**) and immobility time in the forced swim test (**C**-**D**) in males (**A**, **C**) and females (**B**, **D**) subjected to CLP-induced sepsis (*n* = 10-12/group). Habituation memory was evaluated by comparing the number of crossings in the open field between training and test sessions performed 24h apart. Significant reduction in crossings indicates preserved memory. Increased immobility time in the forced swim test indicates depressive-like behavior. Groups: sal+sham, sal+CLP, LPS+sham, and LPS+CLP. Data expressed as mean ± SD. Analysis for habituation memory: paired t-test (training vs. test); for forced swim test: Mann-Whitney test. *p < 0.05 vs. training session or sal+sham group

In the habituation test (number of crossings), animals in the sal+CLP group (males – Figure 3A; females – Figure 3B) did not show a significant reduction in the number of crossings from training to testing, indicating impaired habituation memory. In males, a decrease in crossings during the test compared to training was observed in the sal+sham, LPS+CLP, and LPS+sham groups, indicating memory acquisition. In females, no significant difference was found in the LPS+sham group between training and testing, suggesting that LPS alone was capable of inducing memory impairment. However, in the sal+sham and LPS+CLP groups, females showed reduced crossings in the test compared to training, indicating acquisition of habituation memory. In the habituation test (number of crossings), animals in the sal + CLP group (males – Fig. 3A; females – Fig. 3B) did not show a significant reduction in the number of crossings from training to testing, indicating impaired habituation memory. In males, a decrease in crossings during the test compared to training was observed in the sal+sham, LPS + CLP, and LPS+sham groups, indicating memory acquisition. In females, no significant difference was found in the LPS+sham group between training and testing, suggesting that LPS alone was capable of inducing memory impairment. However, in the sal+sham and LPS + CLP groups, females showed reduced crossings in the test compared to training, indicating acquisition of habituation memory. 

### Sex-specific behavioral responses to sepsis: effects on depressive-like phenotypes

In the forced swimming test, no significant differences in immobility time were found between male groups (Fig. 3C). In females (Fig. 3D), the sal + CLP group displayed increased immobility time compared to the sal+sham group, suggesting depressive-like behavior. LPS + CLP decreased the immobility time compared with sal + CLP group in females. 

### Neuroimmune alterations following CLP and their modulation by prenatal LPS

**Fig. 4:**
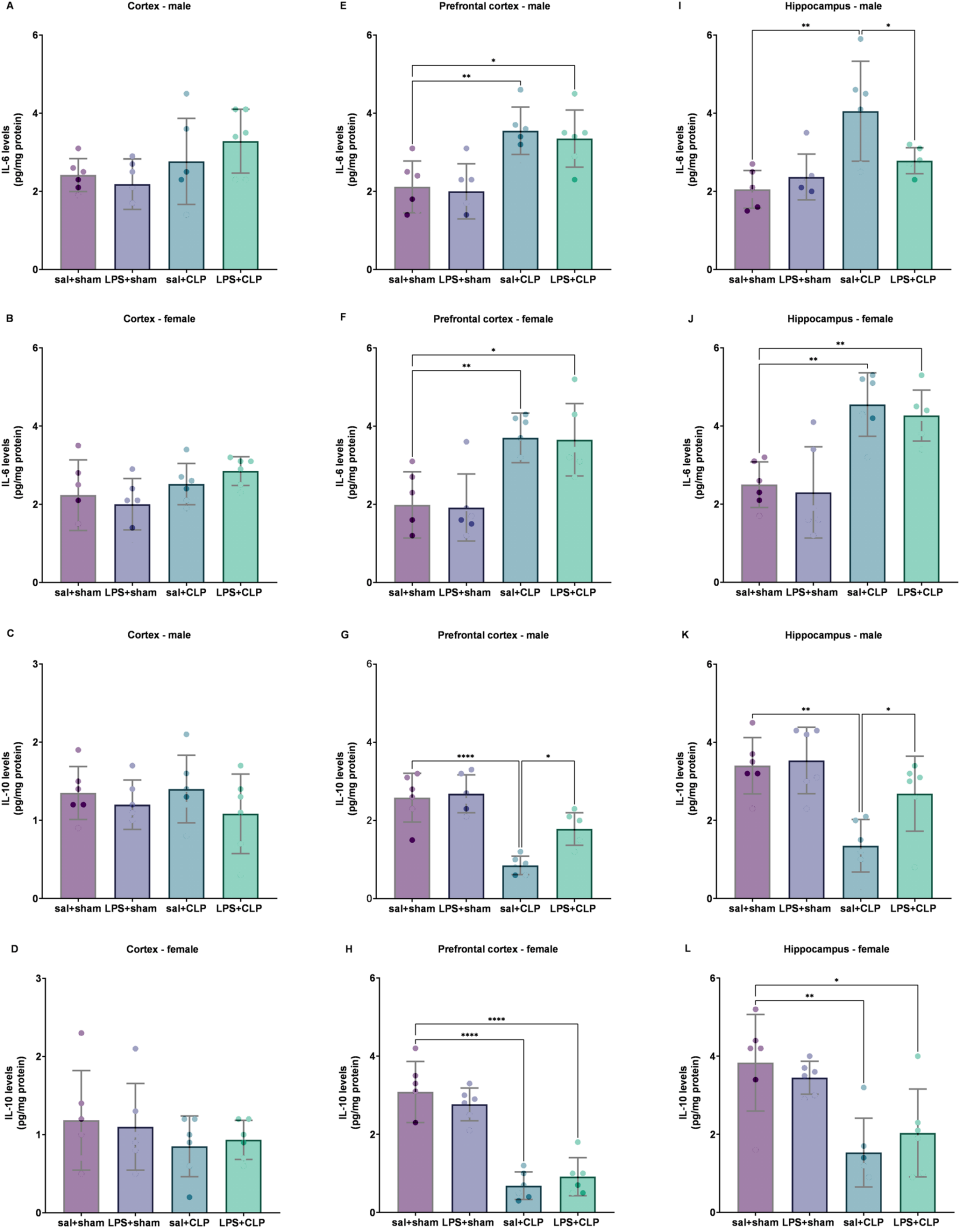
Cytokine levels (IL-6 and IL-10). Quantification of pro-inflammatory IL-6 (**A**, **B**, **E**, **F**, **I **and **J**) and anti-inflammatory IL-10 (**C**, **D**, **G**, **H**, **K **and **L**) in the cortex (A, C: males; B, D: females), prefrontal cortex (E, G: males; F, H: females), and hippocampus (I, K: males; J, L: females) of rats preconditioned with LPS and subjected to CLP-induced sepsis (*n* = 6-8/group). IL-6 is associated with neuroinflammation during sepsis, while IL-10 exhibits neuroprotective effects by modulating the inflammatory response. Groups: sal+sham, sal+CLP, LPS+sham, and LPS+CLP. Data expressed as mean ± SD in pg/mg protein. Analysis: two-way ANOVA followed by Tukey post-hoc test. *p < 0.05, **p < 0.01, ****p < 0.0001

For IL-6 levels in the cortex (Figure 4A and B), no significant differences were detected between groups in either sex. In the prefrontal cortex, IL-6 levels were increased in the sal+CLP and LPS+CLP groups in both males (Figure 4E) and females (Figure 4F) compared to sal+sham. In the hippocampus, IL-6 levels were elevated in the sal+CLP group (males and females – Figures 4I and 4J) versus sal+sham. In males, the LPS+CLP group showed reduced IL-6 levels compared to the sal+CLP group.Regarding IL-10, no significant changes were observed in the cortex of males (Figure 4C) or females (Figure 4D). In males, the sal+CLP group exhibited reduced IL-10 levels in the prefrontal cortex (Figure 4G) and hippocampus (Figure 4K) compared to sal+sham, and this reduction was reversed in the LPS+CLP group. In females, both the sal+CLP and LPS+CLP groups displayed decreased IL-10 levels in the prefrontal cortex (Figure 4H) and hippocampus (Figure 4L) compared to sal+sham. 

### Prenatal LPS modulates sepsis-induced changes in myeloperoxidase activity

**Fig. 5:**
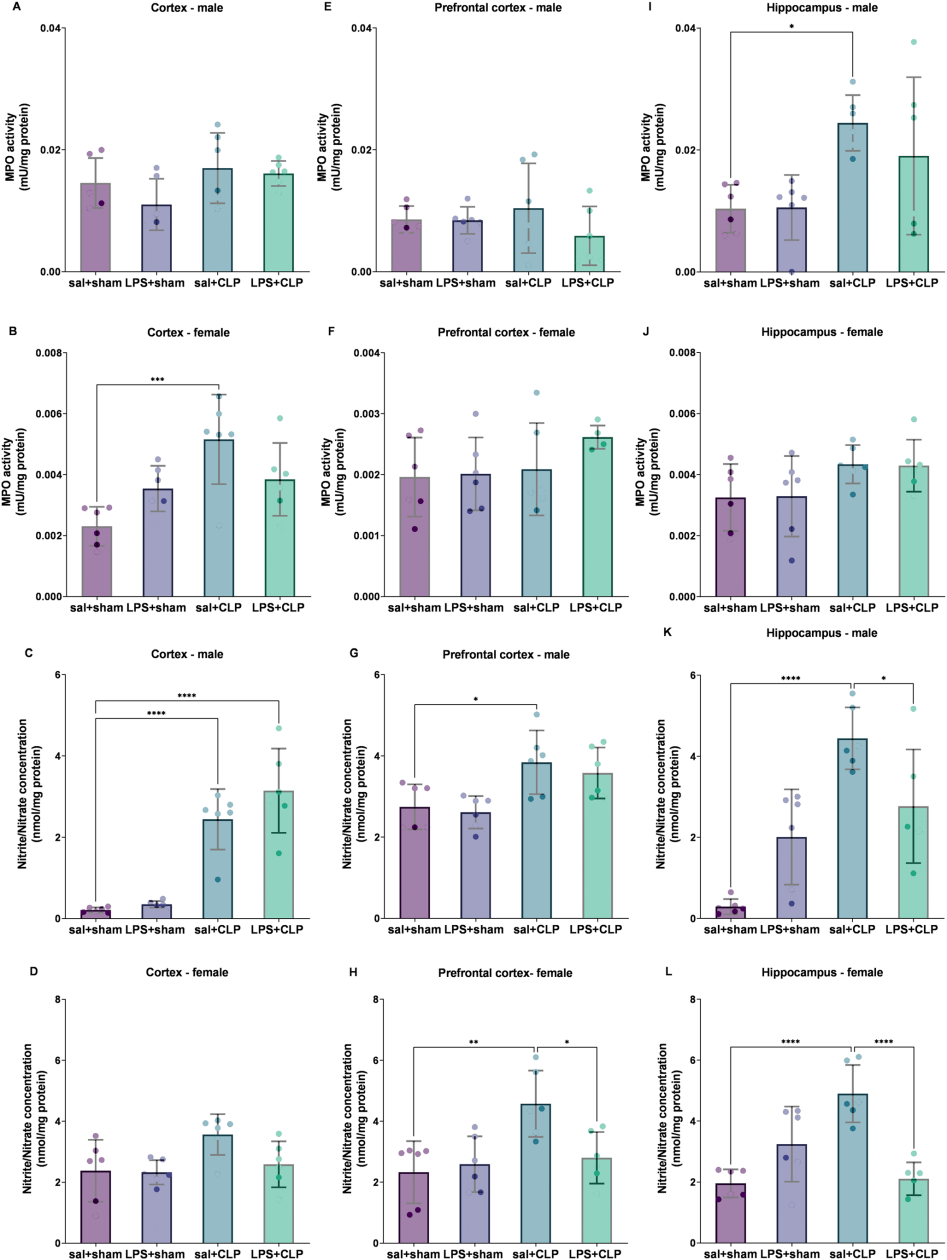
Neuroinflammatory markers: myeloperoxidase activity and nitrite/nitrate concentration. Evaluation of myeloperoxidase (MPO) activity (**A**, **B**, **E**, **F**, **I **and **J**) and nitrite/nitrate levels (**C**, **D**, **G**, **H**, **K **and **L**) in the cortex (A, C: males; B, D: females), prefrontal cortex (E, G: males; F, H: females), and hippocampus (I, K: males; J, L: females) of rats preconditioned with LPS and subjected to CLP-induced sepsis (n = 6-8/group). MPO is a marker of neutrophil infiltration and acute inflammation. Nitrite/nitrate are stable metabolites of nitric oxide, serving as markers of nitrosative stress. Groups: sal+sham, sal+CLP, LPS+sham, and LPS+CLP. Data expressed as mean ± SD in U MPO/mg protein or μM/mg protein. Analysis: two-way ANOVA + Tukey. *p < 0.05, **p < 0.01, ***p < 0.001, ****p < 0.0001

MPO activity was increased in the hippocampus of males (Fig. 5I) and in the cortex of females (Fig. 5B) in the sal + CLP group compared to sal+sham.

### Nitrosative stress markers reveal sex- and region-dependent vulnerabilities

In the cortex, nitrite/nitrate concentrations were elevated in males from both the sal + CLP and LPS + CLP groups relative to sal+sham (Fig. 5C). In females, no statistically significant differences were observed between groups (Fig. 5B). In the prefrontal cortex, males from the sal + CLP group showed increased nitrite/nitrate concentrations compared to sal+sham (Fig. 5G). In females, an increase was observed in the sal + CLP group compared to sal+sham, which was reversed in the LPS + CLP group (Fig. 5H). In the hippocampus of males, nitrite/nitrate concentrations were elevated in sal + CLP group compared to sal+sham and reverted in LPS + CLP group (Fig. 5K). In females, increases were detected in the sal + CLP group compared to sal+sham, which were also reversed in the LPS + CLP group (Fig. 5F). 

###  Oxidative damage patterns reveal differential effects of CLP and prenatal LPS

#### Lipid peroxidation across brain regions

**Fig. 6:**
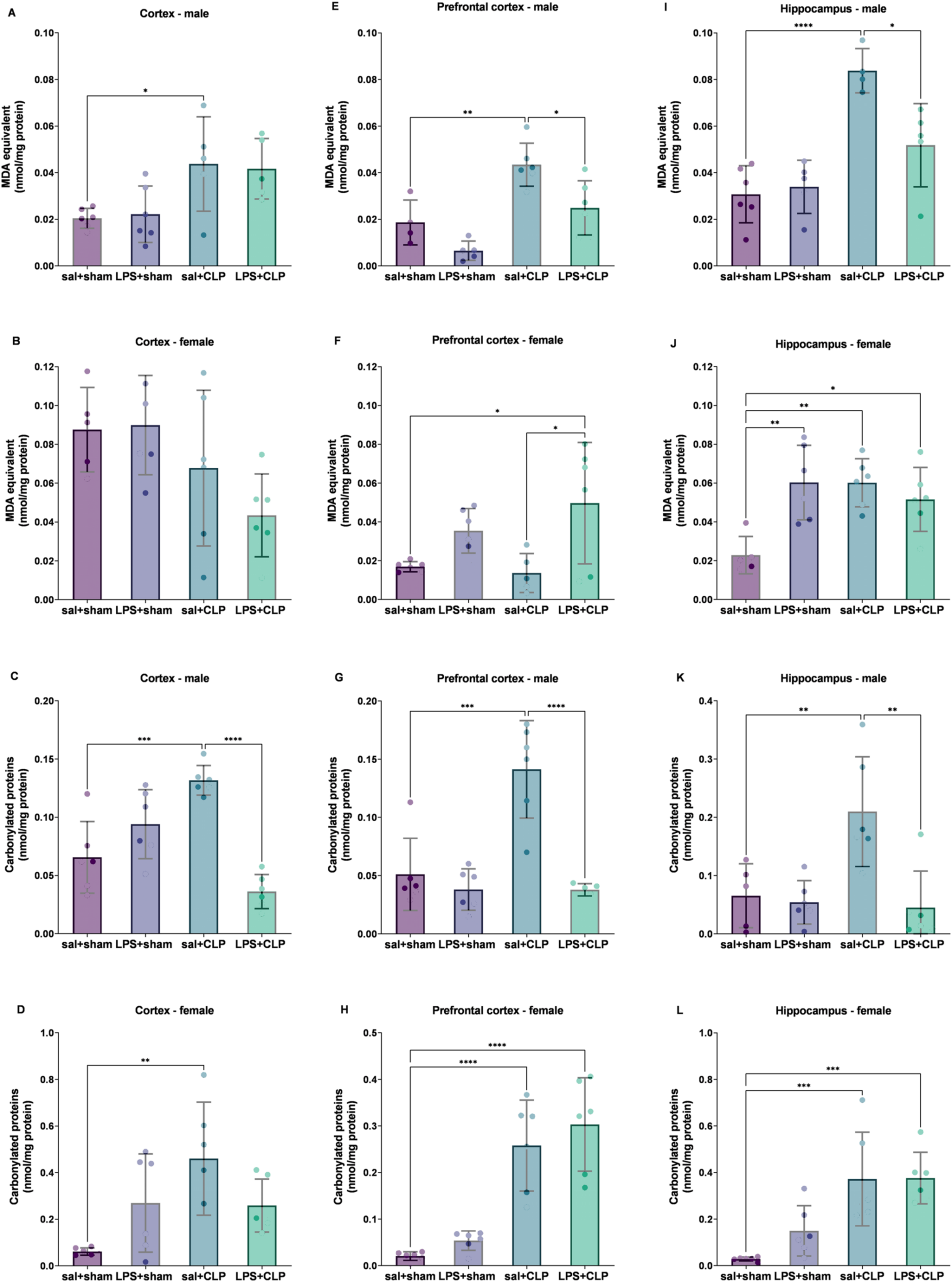
Oxidative damage to lipids and proteins. Quantification of lipid peroxidation via TBARS (**A**, **B**, **E**, **F**, **I **and **J**) and protein carbonylation (**C**, **D**, **G**, **H**, **K **and **L**) in the cortex (A, C: males; B, D: females), prefrontal cortex (E, G: males; F, H: females), and hippocampus (I, K: males; J, L: females) of rats preconditioned with LPS and subjected to CLP-induced sepsis (*n* = 6-8/group). TBARS measures malondialdehyde (MDA) formation as a marker of oxidative lipid damage. Protein carbonylation represents irreversible oxidative protein damage. Groups: sal+sham, sal+CLP, LPS+sham, and LPS+CLP. Data expressed as mean ± SD in nmol MDA/mg protein (TBARS) or nmol carbonyl/mg protein (carbonylation). Analysis: two-way ANOVA + Tukey. *p < 0.05, **p < 0.01, ***p < 0.001, ****p < 0.0001

Lipid oxidative damage was increased in males in the cortex in the sal + CLP group compared to sal+sham (Fig. 6A), whereas no significant differences were observed in females (Fig. 6B). In the prefrontal cortex of males, lipid oxidative damage was elevated in the sal + CLP group relative to sal+sham, but this effect was reversed in the LPS + CLP group (Fig. 6E). In females, an increase was detected in the LPS + CLP group compared to both sal+sham and sal + CLP (Fig. 6F). In the hippocampus of males, the sal + CLP group exhibited increased lipid damage compared to sal+sham, while this effect was reversed in the LPS + CLP group (Fig. 6I). In females, lipid oxidative damage was elevated in the LPS+sham, sal + CLP, and LPS + CLP groups compared to sal+sham (Figure 6J).

#### Protein oxidation (carbonylation) as an index of redox imbalance

Protein oxidative damage (carbonylation) was increased in all brain regions of males in the sal + CLP group compared to sal+sham (Fig. 6C, G, and K). Treatment with LPS + CLP reduced protein oxidation in all evaluated regions compared to sal + CLP. In females, sal + CLP increased protein oxidation in all analyzed structures compared to sal+sham (Fig. 6D, H, and L), with LPS + CLP further enhancing this effect in the prefrontal cortex and hippocampus. 

#### Antioxidant enzyme adaptations: catalase activity across sexes and brain regions

**Fig. 7:**
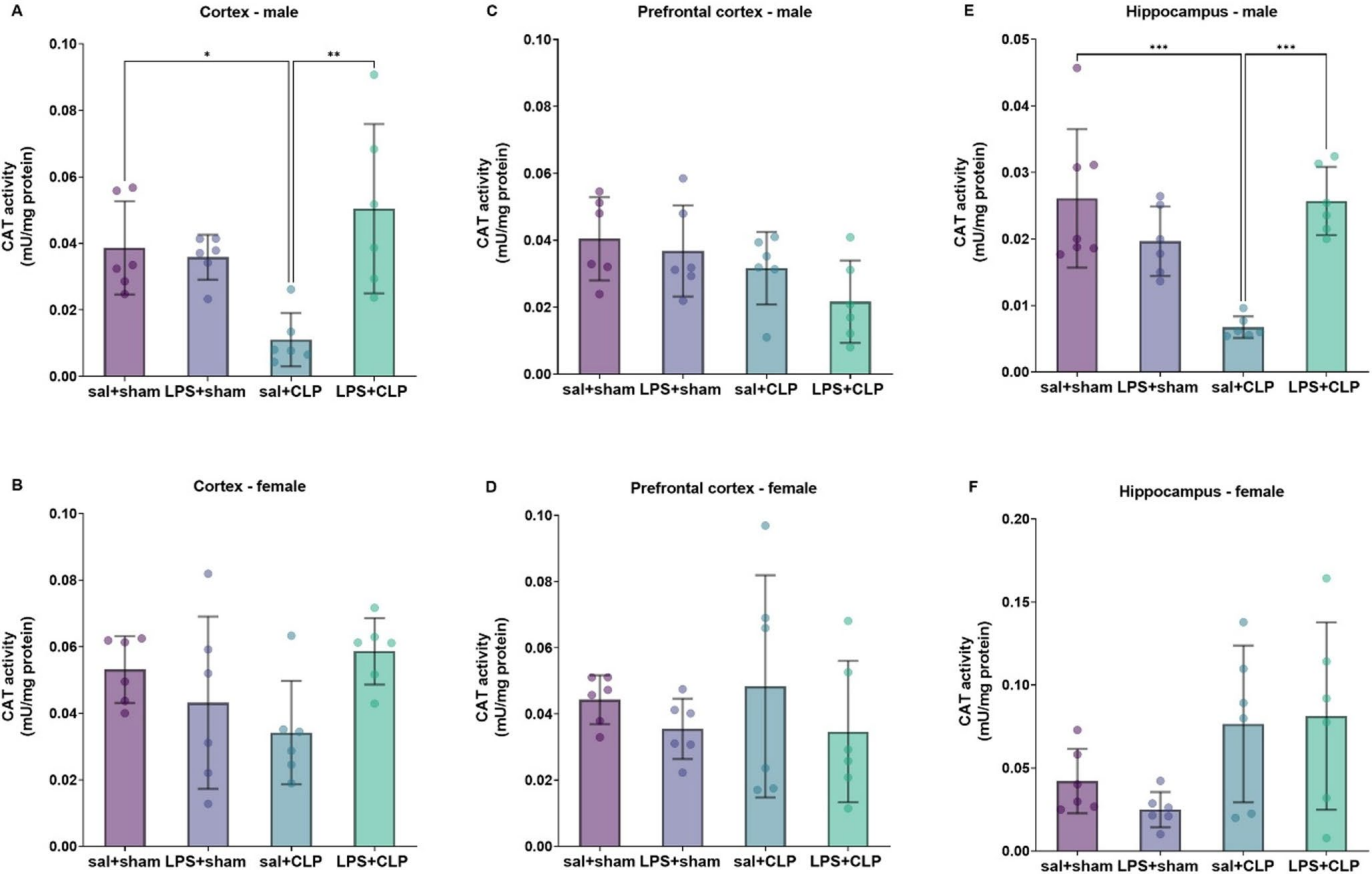
Catalase activity. Determination of the antioxidant enzyme catalase activity (A-F) in the cortex (**A**: males, **B**: females), prefrontal cortex (**C**: males, **D**: females), and hippocampus (**E**: males, **F**: females) of rats preconditioned with LPS and subjected to CLP-induced sepsis (*n* = 6-8/group). Catalase decomposes H₂O₂ into water and oxygen, preventing oxidative stress. Groups: sal+sham, sal+CLP, LPS+sham, and LPS+CLP. Data expressed as mean ± SD in U/mg protein. Analysis: two-way ANOVA + Tukey. *p ≤ 0.05, **p ≤ 0.01, ***p ≤ 0.001, ****p ≤ 0.0001

CAT activity was reduced in the cortex (Fig. 7A) and hippocampus (Fig. 7E) of males in the sal + CLP group compared to sal+sham, and this effect was reversed in the LPS + CLP group. No significant differences in CAT activity were observed in the prefrontal cortex of males (Fig. 7C) or in any of the evaluated brain regions of females (Figure 7B, D, and F). 

### Mitochondrial respiratory chain responses to sepsis and prenatal LPS preconditioning

#### Complex I activity and regional vulnerability

**Fig. 8:**
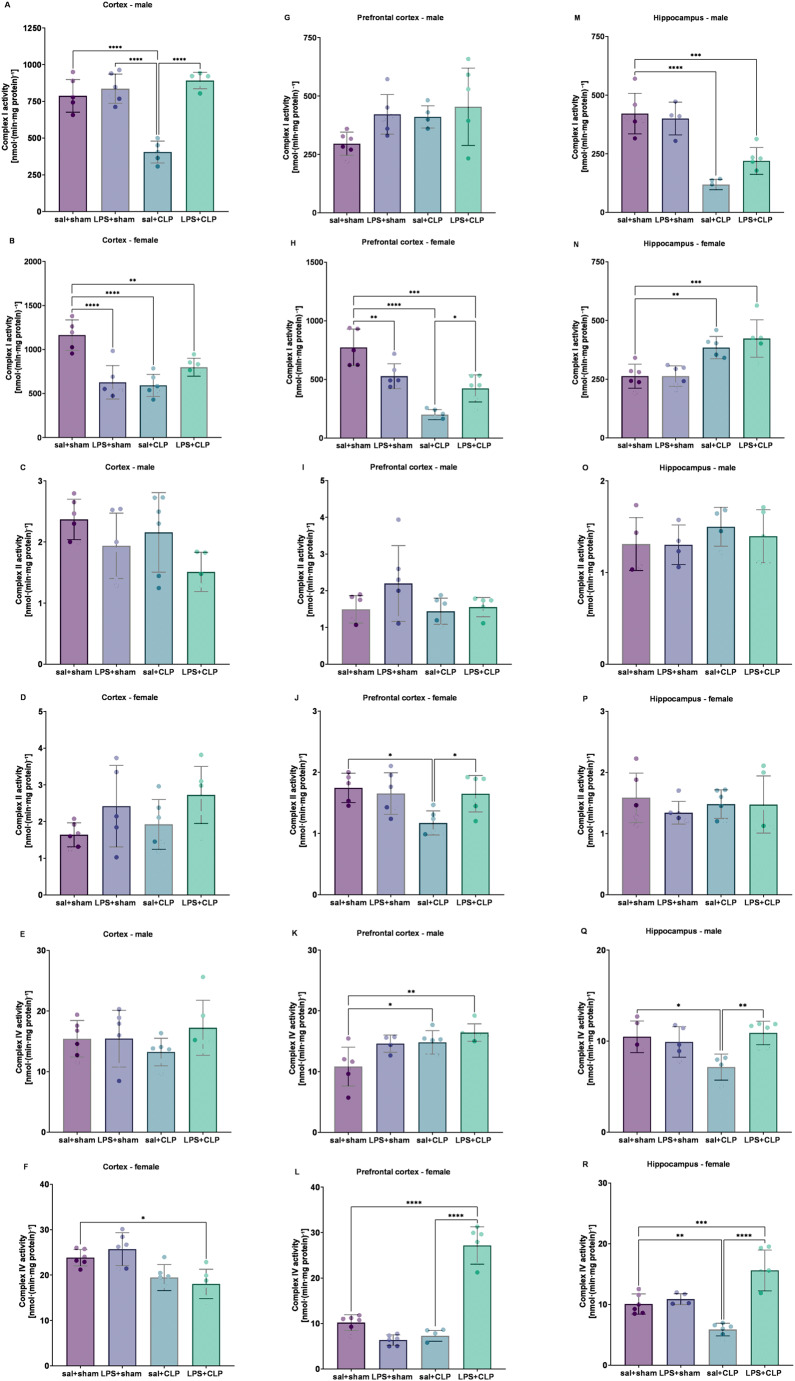
Mitochondrial respiratory chain complex activities (I, II, and IV). Evaluation of mitochondrial complex I/NADH dehydrogenase (**A**, **B**, **G**, **H**, **M **and **N**), complex II/succinate dehydrogenase (**C**, **D**, **I**, **J**, **O **and **P**), and complex IV/cytochrome c oxidase (**E**, **F**, **K**, **L**, **Q **and **R**) activities in the cortex (A, C, E: males; B, D, F: females), prefrontal cortex (G, I, K: males; H, J, L: females), and hippocampus (M, O, Q: males; N, P, R: females) of rats preconditioned with LPS and subjected to CLP-induced sepsis (*n* = 6-8/group). These complexes are essential components of mitochondrial oxidative phosphorylation and energy production. Complex I transfers electrons from NADH to ubiquinone, complex II participates in both the respiratory chain and Krebs cycle, and complex IV is the terminal enzyme transferring electrons to oxygen. Groups: sal+sham, sal+CLP, LPS+sham, and LPS+CLP. Data expressed as mean ± SD in nmol/min/mg protein. Analysis: two-way ANOVA + Tukey. *p < 0.05, **p < 0.01, ***p < 0.001, ****p < 0.0001

Complex I activity remained unchanged in the prefrontal cortex (Figure 8G) of males but was reduced in the cortex (Fig. 8A) of the sal + CLP group and hippocampus (Fig. 8M) of both sal + CLP and LPS + CLP groups compared to sal+sham. In cortex, LPS + CLP increased the level compared with sal + CLP group. In females, complex I activity was decreased in the cortex (Fig. 8B) and prefrontal cortex (Fig. 8H) in the LPS+sham, sal + CLP, and LPS + CLP groups compared to sal+sham. However, in the prefrontal cortex, LPS + CLP increased activity compared to sal + CLP. In the hippocampus (Fig. 8N), females showed increased activity in the sal + CLP and LPS + CLP groups relative to sal+sham.

#### Stability of complex ii under septic and prenatal immune challenge

Complex II activity showed no significant changes in any of the brain regions in males (Fig. 8C, I and O), or in the cortex (Fig. 8D) or hippocampus (Fig. 8P) of females. Only in the prefrontal cortex of females (Fig. 8J) was a reduction observed in the sal + CLP group compared to sal+sham, which was reversed by LPS + CLP.

#### Complex IV modulation reflecting sex- and region-specific mitochondrial adaptation

Complex IV activity was decreased in the cortex of females in LPS + CLP group compared with sal+sham (Fig. 8F). In males, an increase was observed in the prefrontal cortex for both sal + CLP and LPS + CLP groups compared to sal+sham (Fig. 8K). In females, the LPS + CLP group showed increased activity in the prefrontal cortex (Fig. 8L) compared to both sal+sham and sal + CLP. In the hippocampus, activity was decreased in both sexes in the sal + CLP group (Fig. 8Q and R) versus sal+sham, as well as in females in the LPS + CLP group compared to sal+sham. When comparing LPS + CLP to sal + CLP, increased complex IV activity was observed only in the hippocampus of both sexes.

## Discussion

The present study demonstrates that sepsis induced by CLP promotes marked neuroinflammatory, oxidative, and behavioral alterations in offspring, with notable sex-dependent differences. Survival rates were significantly reduced in both males and females, and cognitive assessment revealed that CLP impaired habituation memory, particularly in females exposed to prenatal LPS, while depressive-like behavior was observed predominantly in female offspring. At the molecular level, CLP triggered increased IL-6 and decreased IL-10 in specific brain regions, accompanied by enhanced oxidative damage to lipids and proteins, alterations in antioxidant enzyme activity, and changes in mitochondrial complex function. Interestingly, prenatal exposure to LPS modulated several of these parameters, in some cases attenuating CLP-induced damage such as reducing oxidative injury in males while in others exacerbating deficits, as observed in female prefrontal cortex lipid oxidation. These findings highlight the complex interplay between prenatal immune activation and postnatal septic insult, reinforcing the importance of considering sex-specific responses when evaluating neuroimmune outcomes after sepsis. To our knowledge, this is the first study to evaluate these effects of prenatal LPS exposure combined with adult sepsis induction in both male and female offspring.

Epidemiological data indicate that sex influences both the incidence and outcomes of sepsis. While men exhibit a higher incidence of sepsis, some studies paradoxically report increased mortality among women (Antequera et al. 2021). The underlying mechanisms for these discrepancies remain unclear but may involve sex-based differences in disease presentation, healthcare delivery, or biological factors such as the modulatory role of sex hormones. Androgens have been shown to exert immunosuppressive effects, whereas estrogens exhibit immunoprotective properties (Taneja 2018; Márquez et al. 2020). Supporting this, studies in rats revealed lower sepsis mortality during proestrus compared to ovariectomized animals or males, with improved survival following estrogen receptor agonist treatment (Min et al. 2024). These protective effects of estrogen are attributed to the downregulation of pro-inflammatory cytokines, enhanced organ perfusion, improved cardiac function, and reduced reactive oxygen species production (Faltas et al. 2020; Cassavaugh et al. 2025). Consistently, our study observed higher mortality in male offspring, reinforcing the hypothesis of estrogen’s protective role in sepsis models.

 Beyond high mortality, clinical evidence shows that sepsis survivors frequently experience cognitive deficits (Pan et al. 2017; Kattlun et al. 2024; Ackermann et al. 2025). Memory, a critical aspect of cognition, relies on the integrity of the hippocampus, a brain region vulnerable to sepsis-induced damage (Blair et al. 2013; Wu et al. 2017; Cui et al. 2021). Studies report that sepsis can lead to both acute and chronic cognitive and structural brain damage in adults and in children, where it is associated with poor academic performance (Sewell et al. 2021; Dumbuya et al. 2023). Patients with preexisting cognitive or emotional disorders are particularly susceptible to further impairment; for example, sepsis increases the risk of cognitive decline in elderly patients with depressive symptoms (Battle et al. 2015; Calsavara et al. 2018, 2021).

Preclinical studies support these clinical findings. (Danielski et al. 2018b) demonstrated cognitive impairment in sepsis-surviving animals. Similarly, the CLP sepsis model replicates behavioral deficits observed in patients (Petronilho et al. 2012). The first experimental evidence of learning and memory deficits following physical recovery from CLP-induced sepsis was reported by Barichello et al. (Barichello et al. 2005), with subsequent studies confirming recognition memory deficits and depressive-like behaviors (Barichello et al. 2019; Dal-Pizzol et al. 2021). Memory impairments persisted up to 30 days post-sepsis (Tuon et al. 2008) but were no longer evident at 60 days (Comim et al. 2011). Additional studies reported anhedonia and sleep disturbances in septic animals as well as cognitive and emotional deficits in mice exposed to endotoxemia, by LPS exposure (Baracchi et al. 2011; Anderson et al. 2015). 

Interestingly, while exposure to high doses of LPS is well known to trigger systemic inflammation and long-term cognitive dysfunction, experimental evidence suggests that LPS can also act as an immunological preconditioning agent to second hit. This duality appears to be strongly dependent on dose, timing, and developmental stage of exposure. During early life, low-dose LPS administration has been reported to prime the immune system, enhancing tolerance to subsequent inflammatory insults (Lin et al. 2009). Such preconditioning involves mechanisms of endotoxin tolerance, characterized by a reduced production of pro-inflammatory cytokines upon re-exposure, alongside an increase in anti-inflammatory mediators and regulatory pathways (Seeley and Ghosh 2017; Sangaran et al. 2021). Endotoxin tolerance involves transcriptional reprogramming that downregulates pro-inflammatory cytokine genes and acute-phase proteins. This desensitization also triggers the release of endogenous corticosteroids and IL-10, reduces TLR and MHC expression, alters intracellular signaling, and destabilizes cytokine mRNA (Liu et al. 2019). When the immune system is still maturing, these effects may be particularly relevant, potentially influencing vulnerability or resilience to later septic events (Florentino et al. 2020).

Beloosesky et al(Beloosesky et al. 2010) showed that a single maternal LPS injection during gestation attenuated offspring immune responses to LPS, affecting both pro- and anti-inflammatory pathways. Mechanisms may include reduced monocyte numbers, lower TLR4 expression, or diminished cellular activation. Early fetal TLR4 activation might induce long-term endotoxin tolerance, facilitating microbial colonization postnatally and supporting immune homeostasis (Levy 2007). Consistent with these findings, our study demonstrated that both male and female offspring pre-exposed to prenatal LPS acquired habituation memory following sepsis, suggesting prenatal LPS modulated immune and CNS responses to subsequent severe infectious challenges. 

Similar to cognitive deficits, depressive-like behaviors have been reported in sepsis survivors (Comim et al. 2011; Wang et al. 2021) found that CLP-surviving rats displayed anhedonia, weight loss, elevated corticosterone and ACTH, and reduced hippocampal weight and BDNF. Such depressive-like symptoms mirror those observed in ICU sepsis survivors (Boede et al. 2021). Our study corroborates these findings, showing depressive-like behavior in female offspring following CLP, but not in males. Estrogen’s influence on HPA axis activity stimulating CRH production while androgens inhibit it (Sheng et al. 2021; Handa et al. 2022) may partly explain sex differences in stress responses. Females exhibit higher baseline corticosterone and sex hormones also modulate microglial and astrocyte roles in brain sexual differentiation (Lenz and McCarthy 2015). Given the hippocampus’s role in stress and mood regulation (Fanselow and Dong 2010) these factors may underlie the depressive-like behavior observed in female offspring. Notably, prenatal LPS preconditioning prevented depressive-like behavior in female, suggesting a protective, tolerance-inducing effect.

The improvement observed in habituation memory in males and the attenuation of depressive-like behavior in females from the LPS + CLP groups appear to be directly related to the immunomodulatory effects of prenatal LPS exposure. Literature has shown that repeated LPS exposures can suppress pro-inflammatory signaling by multiple mechanisms. Reductions in TLR4 expression and MyD88-TLR4 association (Chang et al. 2014; Lajqi et al. 2019), decreased IRAK-M activity (Stark et al. 2016), reduced phosphorylation of mitogen-activated protein kinases p38 and c-Jun N-terminal kinase (JNK), and downregulation of NF-κB expression have all been reported (Stark et al. 2016; Yu et al. 2018). In addition, LPS-induced tolerance has been shown to protect against or prevent behavioral alterations in rodents (Banasikowski et al. 2015; Musaelyan et al. 2018).

Elevated IL-6 levels, along with sustained glial activation, have been implicated in increased apoptosis and memory deficits in the hippocampus and prefrontal cortex of sepsis-surviving animals (Catalão et al. 2017, 2020b). Similarly, healthy young humans exposed to endotoxin exhibited transient impairments in episodic and working memory, correlated with increased IL-6 levels (Lozano-Vicario et al. 2023). 

 IL-6 is one of the central mediators of the septic response. It is rapidly synthesized and released by activated macrophages in response to bacterial toxins such as LPS and contributes to the early hyperinflammatory phase of sepsis (Jarczak et al. 2021). High systemic IL-6 levels correlate with disease severity and poor prognosis in both clinical and experimental sepsis, reflecting an exacerbated pro-inflammatory response that drives organ dysfunction (Song et al. 2019). In contrast, IL-10 is a potent anti-inflammatory cytokine that suppresses the synthesis of pro-inflammatory mediators, reduces the expression of adhesion molecules, and downregulates antigen presentation (Peñaloza et al. 2015). This immunoregulatory role allows for pathogen clearance without triggering excessive inflammation or collateral tissue injury. Importantly, sepsis pathophysiology is characterized by a biphasic immune response, with an initial hyperinflammatory state followed by a compensatory anti-inflammatory phase. Reduced IL-10 levels, as observed in the present study, may reflect a predominance of the pro-inflammatory phase at the time of assessment, consistent with reports that dysregulation of this balance exacerbates neuronal and systemic injury (Giustina et al. 2022). 

Interestingly, our results demonstrated that prenatal LPS preconditioning attenuated the sepsis-induced increase in IL-6 exclusively in the hippocampus of male offspring, while also enhancing IL-10 expression in both the hippocampus and prefrontal cortex. These findings support the concept of immune preconditioning and are consistent with a previous study showing that acute or chronic LPS pre-estimulation of primary microglia cultures and organotypic hippocampal slices induced a downregulation of pro-inflammatory genes like IL-6 when being re-stimulated with LPS after 7 days (Antonietta Ajmone-Cat et al. 2013). The sex-dependent differences observed here suggest that such immune reconditioning operates under the influence of sex hormones: estrogens are known to enhance IL-10 release and regulatory T cell activity, whereas androgens are linked to greater susceptibility to hyperinflammation (Klein and Flanagan 2016; Taneja 2018). In males, prenatal LPS exposure appears to have promoted a more effective tolerogenic reprogramming, leading to reduced IL-6 and enhanced IL-10 in response to CLP, consistent with behavioral protection. In contrast, females displayed a less consistent molecular profile, possibly due to the dual role of estrogens, which can be protective in some contexts but may also amplify immune activation depending on dose and receptor pathway. Together, these results indicate that prenatal immune activation functions as a reconditioning event that shapes the trajectory of neuroinflammatory responses to a second septic insult, in a manner tightly modulated by sex-specific molecular mechanisms. 

At the cellular level, shows (Nikodemova et al. 2024) that early-life LPS imprints sex- and region-dependent microglial programs that become evident only after a second inflammatory challenge; this dovetails with our prenatal LPS to adult sepsis findings. Together, they support a model of innate immune “memory” in which developmental LPS exposure establishes neuroimmune set-points that differentially modulate downstream cascades in males and females. In our study, this imprinting aligns with lower hippocampal IL-6 and restored IL-10 in males, paralleling protection of habituation memory whereas benefits in females are more region-restricted. 

Furthermore, prenatal immune stimulation in may contribute to the attenuation of oxidative stress. A recent study in swine showed that prenatal immune stimulation with low-dose LPS can “train” the developing immune system, such that after birth the offspring display a more reactive intestinal immune profile and a shifted oxidative balance. Following a standardized challenge at weaning, prenatal immune stimulation offspring demonstrated greater mucosal immune activation alongside tissue-selective redox changes suggesting enhanced local protection in the gut despite a transient reduction in systemic antioxidant capacity (Mitchell et al. 2025).

Preclinical studies have shown that increased levels of nitric oxide (NO), lipid peroxidation, and protein carbonylation occur in the brain during the early phase of sepsis (at 6, 24, and 48 h post-insult), contributing to acute neural injury (Barichello et al. 2006; Della Giustina et al. 2017b). Furthermore, evidence suggests that oxidative damage may persist long-term, with markers of lipid and protein oxidation detected up to 10, 30, and 60 days following sepsis induction (Steckert et al. 2014).

Interestingly, prenatal LPS exposure modulated these redox alterations in a sex-dependent manner. In males, hippocampal nitrite/nitrate elevations induced by CLP were reversed in the LPS + CLP group, suggesting that immune preconditioning may have limited NO overproduction and subsequent oxidative damage in memory-related structures. In females, the LPS + CLP group showed reversal of nitrite/nitrate increases in both the hippocampus and prefrontal cortex, highlighting a protective role of prenatal LPS in brain areas linked to cognition and emotional regulation. 

In males, the reversal of CLP-induced nitrite/nitrate elevations by prenatal LPS preconditioning was paralleled lower oxidative injury, which otherwise promotes lipid peroxidation, protein carbonylation and inactivates CAT antioxidant enzyme. By dampening upstream pro-inflammatory signaling (lower IL-6) and boosting IL-10, LPS-induced immune preconditioning likely constrained iNOS-driven NO output, thereby limiting ROS/RNS propagation and preserving endogenous defenses (Qin et al. 2016). In males, a higher susceptibility to oxidative stress potentially modulated by testosterone may amplify inflammatory responses and lead to greater consumption of antioxidant enzymes like CAT (Oertelt-Prigione 2012). In contrast, LPS preconditioning may trigger adaptive cellular responses, potentially via activation of antioxidant pathways such as the Nrf2 signaling pathway, which promotes the expression of protective antioxidant proteins (Laskin and Laskin 2001; Laskin 2009). This adaptation could explain the restoration of CAT levels, suggesting that prenatal LPS exposure acts as a protective mechanism, enhancing antioxidant defenses and mitigating oxidative stress-related damage in male offspring, who may be more vulnerable to such injury (de Nooijer et al. 2023). This mechanistic chain IL-6/IL-10 rebalance → lower NO/peroxynitrite → less lipid/protein damage → preserved antioxidant enzyme function offers a coherent explanation for the neuroprotection observed in hippocampus and prefrontal cortex of LPS + CLP males. 

By contrast, in females the LPS preconditioning did not uniformly translate reduced N/N into decreased downstream injury; in some regions, preconditioning coincided with greater lipid/protein damage. This divergence suggests a sex-dependent decoupling between NO surrogates and oxidative end-points, potentially driven by (i) hormone-dependent tuning of TLR4/iNOS/NOX axes and mitochondrial redox set-points, (ii) sex-specific microglial priming and effector programs, and (iii) regionally distinct antioxidant capacity (Réus et al. 2016; Klein and Flanagan 2016; Taneja 2018). Estrogens can bidirectionally modulate inflammatory and redox signaling depending on concentration, receptor context, and cellular state, which may explain why female brains exhibited a more heterogeneous oxidative phenotype after the second hit despite changes in N/N.

In line with a shift toward a less injurious redox milieu, the mitochondrial readouts indicate region- and sex-dependent differences in respiratory resilience. In males, complex I was unchanged in prefrontal cortex but decreased in cortex and hippocampus after CLP; notably, cortical complex I improved in LPS + CLP whereas hippocampal complex I remained reduced. Complex II was largely stable across regions. For complex IV, the hippocampal decrease seen after CLP was accompanied by a relative increase when LPS + CLP was compared with sal + CLP. These patterns are compatible with the known susceptibility of complexes I and IV to nitric oxide/peroxynitrite chemistry and suggest that prenatal LPS limited downstream inhibition of electron transfer at selective sites (Bolaños et al. 1995).

Female profiles were more heterogeneous but still showed region-specific signs of protection: despite a general vulnerability of complex I, selective recovery emerged in prefrontal cortex with LPS + CLP; complex II was mostly unchanged except for a prefrontal dip normalized by LPS + CLP; and complex IV displayed cortical susceptibility alongside LPS-associated gains in prefrontal cortex and hippocampus (particularly when LPS + CLP was compared with sal + CLP). These regional recoveries track with the normalization of nitrite/nitrate in female hippocampus and prefrontal cortex and indicate that prenatal immune training can sustain terminal oxidase function in networks relevant to cognition and affect, even when cortical vulnerability persists.

A parsimonious framework is that prior LPS exposure installs a tolerogenic program that tempers TLR4-driven iNOS output on re-challenge while favoring regulatory/antioxidant pathways, thereby lowering the NO/ONOO⁻ load that compromises complexes I and IV (Liu et al. 2019). The tighter coupling between redox normalization and respiratory recovery in males versus mixed cortical outcomes in females aligns with sexual dimorphism in microglial and mitochondrial tuning by sex hormones, which recalibrate inflammatory and metabolic set-points (Klein and Flanagan 2016; Taneja 2018). 

Therefore, despite some variability in the results, prenatal LPS preconditioning was generally associated with a reduction in exaggerated inflammation, oxidative damage, and mitochondrial dysfunction in both male and female offspring. Nevertheless, further studies are needed to better elucidate the underlying sex differences and to clarify the critical role of prenatal LPS preconditioning in modulating the response to sepsis in adulthood. As limitations of the study, it is important to evaluate changes in the HPA axis, due to its important role in regulating energy metabolism and the immune system. In addition, it would be interesting to evaluate the difference between the sexes and not only between the groups, since there was variability in the findings demonstrated in this study, demonstrating a differentiated response between the sexes.

## Data Availability

No data was used for the research described in the article.
